# Repurposing Therapeutics for Potential Treatment of SARS-CoV-2: A Review

**DOI:** 10.3390/v12070705

**Published:** 2020-06-30

**Authors:** Jennifer Santos, Stephanie Brierley, Mohit J. Gandhi, Michael A. Cohen, Phillip C. Moschella, Arwen B. L. Declan

**Affiliations:** 1 University of South Carolina School of Medicine Greenville, Greenville, SC 29605, USA; jlsantos@email.sc.edu (J.S.); brierles@email.sc.edu (S.B.); mgandhi@email.sc.edu (M.J.G.); 2 Department of Pharmacy, Prisma Health Upstate, Greenville, SC 29605, USA; Michael.Cohen@prismahealth.org; 3 Department of Emergency Medicine Prisma Health Upstate, Greenville, SC 29605, USA; Phillip.Moschella@prismahealth.org

**Keywords:** SARS-CoV-2, COVID-19, coronavirus, repositioning, repurposing, treatment, therapeutics

## Abstract

The need for proven disease-specific treatments for the novel pandemic coronavirus SARS-CoV-2 necessitates a worldwide search for therapeutic options. Since the SARS-CoV-2 virus shares extensive homology with SARS-CoV and MERS-CoV, effective therapies for SARS-CoV and MERS-CoV may also have therapeutic potential for the current COVID-19 outbreak. To identify therapeutics that might be repositioned for treatment of the SARS-CoV-2 disease COVID-19, we strategically reviewed the literature to identify existing therapeutics with evidence of efficacy for the treatment of the three coronaviruses that cause severe respiratory illness (SARS-CoV, MERS-CoV, and SARS-CoV-2). Mechanistic and in vitro analyses suggest multiple promising therapeutic options with potential for repurposing to treat patients with COVID-19. Therapeutics with particularly high potential efficacy for repurposing include camostat mesylate, remdesivir, favipiravir, tocilizumab, baricitinib, convalescent plasma, and humanized monoclonal antibodies. Camostat mesylate has shown therapeutic potential, likely by preventing viral entry into epithelial cells. In early research, the targeted antivirals remdesivir and favipiravir appear to benefit patients by decreasing viral replication; clinical trials suggest that remdesivir speeds recovery from COVID-19. Tocilizumab and baricitinib appear to improve mortality by preventing a severe cytokine storm. Convalescent plasma and humanized monoclonal antibodies offer passive immunity and decreased recovery time. This review highlights potential therapeutic options that may be repurposed to treat COVID-19 and suggests opportunities for further research.

## 1. Introduction

The new pandemic coronavirus disease 2019 (COVID-19) is caused by the novel Severe Acute Respiratory Syndrome coronavirus 2 (SARS-CoV-2), which belongs to the *Coronaviridae* family [1]. The *Coronaviridae* family is a cohort of viruses with single-stranded, positive-sense RNA genomes that typically cause both respiratory and enteric diseases [2]. The three coronaviruses, Severe Acute Respiratory Syndrome coronavirus (SARS-CoV), Middle East Respiratory Syndrome coronavirus (MERS-CoV), and SARS-CoV-2 can cause severe respiratory illnesses, known as SARS, MERS, and COVID-19, respectively. The current pandemic COVID-19 is a primarily respiratory disease with a spectrum of severity ranging from mild upper respiratory illness to acute respiratory distress syndrome, pneumonia, multi-organ failure, and ultimately death [3].

The three coronaviruses SARS-CoV, MERS-CoV, and SARS-CoV-2 all belong to the *betacoronavirus* genus [3]. Sequence analysis of the SARS-CoV-2 genome shows that it is 82% identical to that of SARS-CoV [1], whereas MERS-CoV shares approximately 50% genomic sequence identity with SARS-CoV-2 [4]. While these three viruses are zoonotic in origin, the spread of SARS-CoV and MERS-CoV was generally limited to nosocomial and intra-family transmission, whereas SARS-CoV-2 spreads more rapidly and has achieved widespread community transmission.

Each of these three coronaviruses causes significant morbidity and mortality in humans. In 2003, a SARS-CoV outbreak started in China and caused 8,098 confirmed cases worldwide with 775 fatalities [2]. In 2012, the MERS-CoV outbreak that emerged in Saudi Arabia caused 22,260 confirmed cases with 803 fatalities [2]. The current SARS-CoV-2 pandemic started in China in late 2019 and has caused over 10,000,000 human cases and more than 500,000 fatalities as of June 29th, 2020 [5,6]. The SARS-CoV-2 outbreak has far surpassed that of SARS-CoV and MERS-CoV in magnitude, with the number of cases and deaths increasing daily.

Given the lack of vaccines or specific treatments for the novel coronavirus SARS-CoV-2, it is crucial to identify therapeutic options to both limit the replication of this virus and prevent further spread. Based on the extensive homology shared by SARS, MERS, and COVID-19, we hypothesize that drugs with evidence of benefit in treatment of SARS or MERS are likely to benefit patients with COVID-19. This review summarizes drugs and therapies with either theoretical, *in vitro*, or in vivo antiviral activity against SARS-CoV or MERS-CoV to strategically identify therapeutic options that can be used or repurposed for the treatment of SARS-CoV-2.

## 2. Materials and Methods

PubMed, CINAHL, EMBase, and Google Scholar were searched to identify articles that supported therapeutic benefit for a pharmacological substance in SARS-CoV or MERS-CoV that could be repositioned for treatment of SARS-CoV-2 infections. Four of the authors conducted independent searches to evaluate various search strategies. These combined strategies were shared and optimized with the Prisma Health System staff medical librarians. The aggregate search strategy includes articles limited to those available prior to June 15, 2020. Search criteria included the keywords coronavirus, severe acute respiratory syndrome, Middle East respiratory syndrome, SARS, MERS, SARS-CoV, MERS-CoV, SARS-CoV-2, COVID-19 in conjunction with the terms treatment or therapeutic as well as the following Medical Subject Headings: antiviral agents/therapeutic use, coronavirus infections/drug therapy, coronavirus infections/therapy, drug repositioning, Middle East respiratory syndrome coronavirus, Middle East respiratory syndrome coronavirus/drug effects, SARS virus, SARS virus/drug effects, severe acute respiratory syndrome/drug therapy, and treatment outcome. The initial search strategy identified a total of 353 articles. Next, the names of the individual medications reviewed in this article were also used in conjunction with the above search criteria to identify an additional 99 articles, and CENTRAL was searched for relevant trials. Finally, bibliographic references of the pertinent articles were also searched for relevant literature not identified in the original search strategy. Each article was reviewed by a minimum of two authors to assess suitability for inclusion in this review. The authors reviewed the literature for inclusion based on critical evaluation of the relevance and methods of candidate papers. Articles were accepted for inclusion based on a modified Delphi method.

## 3. Results

### 3.1. Targeted Enzyme Inhibitors

#### 3.1.1. RNA-Dependent RNA Polymerase Inhibitors

The RNA-dependent RNA polymerase (RdRp) enzymes replicate and transcribe the viral genome of RNA viruses, including coronaviruses. Since these essential enzymes are highly conserved across RNA viruses, they are attractive targets for anti-viral therapies. The prodrugs remdesivir and favipiravir inhibit RdRp and are now under evaluation for treatment of COVID-19. Other RdRp inhibitors, such as sofosbuvir, have not been evaluated for activity againstcoronaviruses [7].

##### Remdesivir

Remdesivir is a promising investigational nucleoside analog [8] with potent antiviral activity against coronaviruses [4]. Remdesivir is a prodrug of remdesivir triphosphate (RDV-TP), an adenosine triphosphate (ATP) analog that inhibits the SARS-CoV-2 RdRp enzyme [4]. In the triphosphate form, RDV-TP efficiently competes with ATP for incorporation into the viral RNA. This mechanism causes delayed chain termination 3 to 5 base pairs later [9]. Remdesivir reduced overall virus replication and lung damage in rhesus macaques infected with MERS-CoV [10,11]. It also showed therapeutic efficacy against SARS-CoV in a mouse model [10]. Additionally, remdesivir exhibited potent in vitro antiviral activity against SARS-CoV-2 isolates [12].

Expanded Access or Compassionate Use programs have provided remdesivir to patients with confirmed, severe SARS-CoV-2 infections in the United States, Europe, and Japan [4]. Although initial Chinese results showed no improvement in endpoints including time to clinical improvement [13], preliminary results from the Adaptive Covid-19 Treatment Trial (ACTT-1) showed a statistically significant shorter time to recovery in the treatment arm as compared to the control arm (11 days versus 15 days; rate ratio for recovery, 1.32; 95% confidence interval [CI], 1.12 to 1.55; *p* < 0.001) [14]. Even though remdesivir does not decrease SARS-CoV-2 RNA viral loads or reduce mortality [13], these early results support continued use of remdesivir as well as further research to validate currently available results.

##### Favipiravir

Favipiravir is an RdRp inhibitor that blocks the replication of multiple RNA viruses by terminating elongation at the incorporation site as a nucleoside analogue. Like remdesivir, it functions as an inhibitory RdRp substrate in its phosphorylated form, favipiravir-triphosphate, and inhibits the SARS-CoV-2 RdRp [15]. It is currently approved for influenza treatment in Japan and is well-tolerated by humans [16]. In high concentrations, favipiravir decreases SARS-CoV-2 viral infection in Vero cells [12,15]. A recent open-label, randomized controlled trial found that patients treated with favipiravir plus interferon-alfa cleared SARS-CoV-2 infection more rapidly than patients treated with lopinavir/ritonavir plus interferon-alfa combination therapy. Specifically, lung imaging abnormalities improved more rapidly in the favipiravir treatment group [17]. This early evidence suggests that favipiravir may be a beneficial therapy for SARS-CoV-2.

#### 3.1.2. Neuraminidase Inhibitors

Neuraminidase inhibitors, such as oseltamivir, are used to treat Influenza A and Influenza B [18,19]. The influenza viruses depend on neuraminidase enzymes for viral spread. Neuraminidase inhibitors mimic sialic acid to prevent neuraminidase from cleaving the glycosidic linkage between sialic acid and the glycoprotein, thereby decreasing virion release, preventing viral reproduction, and decreasing the spread of the influenza virus in the respiratory tract of infected individuals when used early in the disease process [19]. Although oseltamivir has been used in several Chinese hospitals for severe or suspected SARS-CoV-2 cases [8,20], it has not been proven effective against SARS-CoV-2 either in vitro or in vivo [21].

#### 3.1.3. Protease Inhibitors

Protease enzymes, which contribute to multiple protein processing, signaling, and degradative cellular pathways by cleaving peptide bonds, are important drug targets across a range of diseases [22,23]. These enzymes catalyze a wide range of physiological processes. While their essential functions make them attractive drug targets, their complex interactions and broad impact often introduce unwanted side effects and poor selectivity. Several currently available protease inhibitors have been identified as potential anti-coronavirus medications.

##### Lopinavir/Ritonavir

Lopinavir is a human immunodeficiency virus (HIV) protease inhibitor that also inhibits SARS-CoV viral replication. Ritonavir, another HIV protease inhibitor, increases lopinavir concentrations by inhibiting the cytochrome P450 3A4-mediated metabolism of lopinavir [24]. Combination therapy with lopinavir/ritonavir (LPV/r) has been studied for treatment and prevention of SARS-CoV and MERS-CoV. SARS-CoV reaches its peak of viral replication in approximately ten days. Theoretically, LPV/r use within this window prevents the viral load from rising to a level that overwhelms the patient’s immune response [24]. Studies in SARS-CoV patients revealed improved outcomes after treatment with LPV/r as compared to controls [24] or after treatment with lopinavir/ritonavir, steroids, and ribavirin as compared to controls treated with ribavirin and steroids [25]. When the LPV/r combination was administered as post-exposure prophylaxis (PEP) in health care workers exposed to MERS-CoV, this combination therapy decreased the risk of MERS-CoV infection by 40% and was well tolerated [24]. Although computational evidence does not show binding between LPV/r and SARS-CoV-2 drug targets such as RdRp, the papain-like protease (PLpro), or the main protease [26], lopinavir inhibits in vitro replication of SARS-CoV-2 [27]. While LPV/r has been used clinically in patients with COVID-19 [27], a randomized, controlled, open-label clinical trial showed no significant mortality benefit of treatment with LPV/r in patients with severe COVID-19 as compared to standard care, though patients treated with LPV/r had shorter lengths of intensive care unit (ICU) stay (secondary outcome: 6 days LPV/r; 11 days standard; delta –5 days; 95% CI, -9 to 0) [28,29]. Thus, additional study of lopinavir/ritonavir therapy as a SARS-CoV-2 treatment is needed to clarify potential benefits.

##### TMPRSS2 Inhibitors

Transmembrane Protease, Serine 2 (TMPRSS2) is a serine protease that is required for SARS-CoV-2 entry into host cells; TMPRSS2 cleaves the viral spike (S) protein to prime it for binding to the host cell angiotensin-converting enzyme 2 (ACE2) [30]. The S protein and ACE2 receptor are highly conserved among the MERS-CoV, SARS-CoV, and SARS-CoV-2 viruses [30]. Thus, TMPRSS2 has been identified as a promising target for treatment of COVID-19 [30]. The TMPRSS2 inhibitors camostat mesylate, nafamostat, and bromhexine may treat COVID-19. Camostat mesylate is used in Japan for the treatment of pancreatitis. Previous in vitro work showed that camostat suppresses MERS-CoV and SARS-CoV infections in cell cultures [30,31]. It is currently under evaluation in a randomized clinical trial as a treatment for SARS-CoV-2. Nafamostat mesylate is another serine protease inhibitor that may block human TMPRSS2. Nafamostat inhibits MERS-CoV infection [32] and was recently shown to interfere with SARS-CoV-2 entry into cells at a low concentration [33]. Bromhexine is a generic mucolytic that inhibits TMPRSS2 and is available outside of Japan [34]. It is currently being investigated clinically as a treatment for SARS-CoV-2. While the TMPRSS2 inhibitors camostat, nafamostat, and bromhexine are very promising treatments currently under investigation, they represent therapies that currently may not be widely available outside of Asia.

##### Disulfiram

Disulfiram inhibits hepatic aldehyde dehydrogenase and is approved by the U.S. Food and Drug Administration (USFDA) for alcohol aversion therapy [35]. In vitro work showed that disulfiram also inhibits the SARS-CoV and MERS-CoV papain-like protease PLpro [35]. The PLpro enzyme participates extensively in host cell control and contributes to viral replication by cleaving the viral polyprotein into functional units. In vitro studies demonstrated that the targeted PLpro inhibitor GRL0617 blocked SARS-CoV replication [36]. While this particular compound is preclinical, this study supports targeting PLpro for anti-coronavirus drug development. PLpro is highly conserved between SARS-CoV and SARS-CoV-2 [26]. Thus, while there is minimal data to support actual efficacy of disulfiram as therapy for COVID-19 [37], there is theoretical support for repurposing.

##### Angiotensin-Converting Enzyme 2 Inhibitors

The angiotensin-converting enzyme 2, or ACE2, belongs to the angiotensin-converting enzyme (ACE) family of dipeptidyl carboxydipeptidases. ACE2 inactivates angiotensin II and generates angiotensin 1–7, a heptapeptide with potent vasodilator function via activation of the Mas receptor. ACE2 performs a vital role in the cardiovascular and immune systems but is used as a functional receptor by SARS-CoV-2 to infect human cells. SARS-CoV and SARS-CoV-2 endocytosis depends on viral spike protein binding to ACE2 [30], which is highly expressed in the heart and lungs [38]. Overexpression of ACE2 in patients with underlying cardiovascular disease may therefore result in severe respiratory symptoms in patients with underlying cardiovascular diseases such as hypertension, cerebrovascular disease or diabetes [39]. Thus, the ACE2 receptor may modulate COVID-19 disease severity.

Although prior structural analyses identified the ACE2 inhibitor *N*-(2-Aminoethyl)-1-aziridineethanamine (NAAE), which inhibits SARS cell fusion *in vitro*, as a potential therapy for SARS [40], this compound has not been developed further. Clinical trials are ongoing, both to guide the use of ACE inhibitors for possible treatment of COVID-19 and for chronic management of patients with hypertension in the context of pandemic COVID-19. Current evidence suggests that ACE inhibitors do not worsen outcomes in COVID-19 and should be continued in stable patients [41].

#### 3.1.4. Kinase Inhibitors

Kinase enzymes transfer phosphate groups from ATP to recipient proteins in a process called phosphorylation. This process transfers physiological signals, often via cascades of sequential reactions. Since cancer often deregulates signaling pathways, protein kinases are key targets for oncological drug development. Similarly, since many viruses hijack host signaling pathways, kinase inhibitors identified in other contexts may be repositioned for use as broad-spectrum or targeted antiviral therapy [42].

##### Imatinib

Imatinib, also known as Gleevec, is an oral tyrosine kinase inhibitor with demonstrated activity against Ebola and poxviruses [43]. Imatinib targets the Abelson tyrosine-protein kinase 2 (Abl2) that is required for SARS-CoV and MERS-CoV replication. In vitro studies showed that imatinib inhibits MERS-CoV and SARS-CoV infection with relatively low cellular toxicity [43,44]. Although results from initial clinical trials are not yet available, mechanistic and in vitro arguments suggest that future clinical research would be useful.

##### Baricitinib

Baricitinib is a Janus kinase (JAK) inhibitor used to treat rheumatoid arthritis [45]. Recent machine learning studies have identified baricitinib as a promising antiviral therapy for SARS-CoV-2 [46]. Baricitinib displays multifactorial activity. In addition to inhibiting JAK, it also binds the adaptor-associated kinase-1 (AAK1) that is essential for clathrin-mediated viral endocytosis. It may also dampen host cytokine release. A recent pilot study compared the clinical impact of baricitinib plus lopinavir/ritonavir to standard COVID-19 therapy. The baricitinib group showed a statistically significant improvement in fever, dyspnea, and hypoxia [47]_._ Since baricitinib decreases viral entry and blunts cytokine storm [48], it is an ideal candidate for further study in SARS-CoV-2.

#### 3.1.5. Ribavirin

Ribavirin is a purine nucleoside analog with antiviral activity that is currently approved for treatment of chronic hepatitis C [49]. As an antiviral, it halts replication of many RNA and DNA viruses by inhibiting the enzyme inosine monophosphate dehydrogenase. Ultimately, this leads to destruction of the RNA genome [49].

Ribavirin inhibits MERS-CoV viral replication when administered as combination therapy with other antivirals [50]. In patients with severe MERS-CoV infection, 70% of patients receiving the combination ribavirin and interferon alfa-2a therapy were alive at 14 days compared to only 29% in the control group [50]. Although ribavirin has antiviral activity against Respiratory Syncytial Virus (RSV), influenza viruses, and parainfluenza viruses *in vitro*, it does not appear to inhibit SARS-CoV [49]. Furthermore, SARS-CoV patients treated with ribavirin and corticosteroids had a marked increase in viral load in their upper respiratory tract during therapy [51]. New evidence in the context of SARS-CoV-2 suggests that ribavirin may inhibit PLpro and may, therefore, decrease viral replication [26]. In a recent open-label, randomized phase 2 clinical trial, patients treated with combination ribavirin, LPV/r and interferon-1b had resolution of viral shedding more quickly than patients treated with LPV/r (median time to negative viral swab, 7 days vs. 12 days control; hazard ratio 4.37; 95% CI 1.86 to 1024) [52]. Unfortunately, ribavirin has significant adverse effects, including hemolytic anemia [53], hypocalcemia, and hypomagnesemia [49,51]; it is under investigation primarily as combination therapy.

### 3.2. Immunomodulators

#### 3.2.1. Convalescent Plasma

Convalescent plasma containing SARS-CoV-2 antibodies is being administered to severely ill patients with COVID-19 as an adjunct immunotherapy based on the hypothesis that the plasma contains neutralizing antibodies for SARS-CoV-2. Previous work showed that antibodies to the S protein were present for more than 7 months in the serum of SARS patients [54]. In a series of severely ill SARS patients who received convalescent plasma, the length of stay and overall mortality of the severely ill patients who received convalescent plasma was less than the contemporaneous local mortality [55]. In two small case series, patients with COVID-19 also improved clinically after treatment with convalescent plasma [56,57]. An open-label, multicenter, randomized clinical trial did not show a significant benefit in time to clinical improvement (percent improved within 28 days, 51.9% vs. 43.1% control; hazard ratio 1.4; 95% CI 0.79–2.49) or the secondary outcome of 28-day mortality (15.7% vs. 24.0% control; odds ratio 0.65; 95% CI 0.29–1.46) in patients with severe COVID-19. However, this study was terminated prematurely and underpowered due to local disease containment prior to full patient enrollment [58]. The USFDA is currently facilitating access to SARS-CoV-2 convalescent plasma for patients with severe COVID-19, and studies are ongoing.

#### 3.2.2. Humanized Antibodies

Human monoclonal antibodies also have robust potential for supporting passive immunity, whether via use as pre-exposure prophylaxis or for treatment of severe cases [59].

##### Spike Protein Antibodies

The humanized antibodies mAb 4C2h and mAb 5H10 are murine antibodies that neutralize the spike protein receptor-binding domain of the MERS and SARS viruses, respectively. In mice, treatment with the mAb 4C2h antibodies significantly decreases MERS-CoV viral titers [60]. The mAb 5H10 antibody decreased viral load and pulmonary damage in a rhesus macaque model of SARS [61]. Monoclonal antibodies may be an effective therapy for SARS-CoV-2. However, their availability depends on either rapid research progress or successful repurposing of antibodies that were previously identified based on SARS-CoV and MERS-CoV homologies.

##### IL-6 Receptor Antibodies

Tocilizumab and sarilumab are humanized monoclonal antibodies specific for the interleukin 6 (IL-6) receptor; both are USFDA approved for the treatment of rheumatoid arthritis [48]. Multiple studies have revealed elevated levels of cytokines, including IL-6, in patients with severe COVID-19 [45,48]. Tocilizumab has been used to treat severely or critically ill SARS-CoV-2 patients with extensive lung lesions and high IL-6 levels [48,62]. In a small, retrospective case series, patients treated with tocilizumab improved over about 5 days, with decreased oxygen requirements, improved laboratory markers of inflammation, and resolution of pulmonary abnormalities. Similarly, 7 of 8 patients treated with sarilumab improved to hospital discharge [62,63]. Since early clinical experiences with these IL-6 inhibitors were sufficiently positive, tocilizumab and sarilumab are both being studied in vitro for treatment of COVID-19.

#### 3.2.3. Interferons

Interferons are cytokines that activate the host immune system and thus broadly combat viral infections. Empirically, several interferons have in vitro antiviral activity [64,65]. Interferon alfacon-1 is a genetically engineered composite synthetic interferon based on the naturally occurring alpha interferons. In a small, non-randomized trial, patients with SARS-CoV treated with interferon alfacon-1 and steroids had better clinical outcomes than patients treated with steroids without interferon alfacon-1 [64]. Other studies have suggested that type 1 interferons benefit patients with SARS-CoV and MERS-CoV [66,67]. Type 1 interferons also inhibit SARS-CoV-2 *in vitro.* Early results of interferon-beta-1b as combination therapy have been encouraging, and trials to evaluate the use of type 1 interferons as adjunctive therapy for SARS-CoV-2 are ongoing [52,68].

#### 3.2.4. Cyclosporine A

Cyclosporine A is a calcineurin inhibitor used as an immunosuppressant and as an antiviral treatment for hepatitis C [69] and MERS-CoV [70]. Previous in vitro studies have shown that cyclosporine A inhibits replication of SARS and other coronaviruses. While its antiviral mechanism of action is not well understood, it is thought to act by inhibiting cyclophilin protein pathways [71,72]. Since cyclosporine A inhibits the growth of all tested coronaviruses, it is potentially beneficial in patients with COVID-19. Unfortunately, its use may be limited by its toxicity. However, a small case series of kidney transplant recipients changed to or maintained on cyclosporine A during treatment for COVID-19 did not show evidence of harm [73].

#### 3.2.5. Mycophenolic Acid

Mycophenolic acid (MPA) is also an immunosuppressant that may have antiviral activity [45]. Although its mechanism of action is not clearly defined, initial in vitro studies suggested that MPA can inhibit the papain-like proteases of both MERS-CoV and SARS-CoV [45]. However, in vivo studies in marmosets with MERS-CoV infection treated with MPA and interferon beta revealed high viral loads with severe and even fatal disease. Furthermore, the small number of MERS patients treated with MPA had worse functional outcomes than patients treated with other antivirals [45]. Thus, despite early promising in vitro studies, MPA does not appear to be an optimal candidate for treatment of COVID-19.

#### 3.2.6. Corticosteroids

Corticosteroids have been investigated extensively as adjunctive viral therapy due to their immunomodulatory effect. Since corticosteroids suppress host inflammatory responses, they offer theoretical benefit against excessive inflammasomal responses. However, this mechanism also suppresses natural defense mechanisms. Thus, corticosteroids may cause more harm and even increase mortality when used as adjuncts to antiviral therapies. When corticosteroids were used as adjunct therapies for treatment of SARS and MERS [74,75], a plethora of significant adverse effects were identified, including prolonged hospitalizations without mortality benefit [76]. One study even demonstrated increased 30-day mortality in SARS patients treated with pulsed methylprednisolone (adjusted odds ratio 26.0, 95% CI 4.4 to 154.8) [77].

Surprisingly, recently released, preliminary, non-peer-reviewed data from the Randomised Evaluation of COVID-19 therapy (RECOVERY) trial described reduced mortality in severely ill patients with COVID-19 who were treated with dexamethasone for 10 days [78]. Although these initial results seem promising, further exploration of the data is warranted before the results are adopted clinically.

### 3.3. Antibiotics

#### 3.3.1. Glycopeptide Antibiotics

Several glycopeptide antibiotics have been studied for treatment of coronaviruses. Teicoplanin is a glycopeptide which is used to treat complicated infections associated with commonly susceptible organisms including aerobic and anaerobic gram-positive bacteria. Teicoplanin is marketed in the United Kingdom but is not available in the United States [79]. Teicoplanin inhibits viral spike protein cleavage by cathepsin L and thus prevents viral replication [80]. The cleavage site that cathepsin L targets is conserved among MERS-CoV, SARS-CoV, and SARS-CoV-2 [81]. In vitro studies revealed that the teicoplanin derivatives dalbavancin and oritavancin inhibit MERS-CoV and SARS-CoV cell entry [81]; dalbavancin decreased entry of SARS-CoV-2, with dose-dependent effects. A case series of 21 patients treated with teicoplanin revealed no adverse effects [82]. Given their in vitro efficacy, glycopeptide antibiotics warrant further investigation for treatment of SARS-CoV-2 infection [80].

#### 3.3.2. Nitazoxanide

Nitazoxanide (NTZ) was originally developed to treat protozoal infections. By inhibiting the pyruvate ferredoxin oxidoreductase enzyme-dependent electron transfer, NTZ uncouples the oxidative phosphorylation pathways of energy metabolism [83]. However, it has also been shown to exert broad spectrum antiviral activity. NTZ’s active metabolite, tizoxanide, has broad-spectrum in vitro antiviral activity; NTZ and tizoxanide both inhibit MERS-CoV and other coronaviruses in vitro [37,83,84]. Murine studies suggest that NTZ also inhibits N protein expression and suppresses pro-inflammatory cytokines [84]. NTZ is being evaluated as a treatment for influenza and appears to be relatively well tolerated [84]. It is thus also a promising therapeutic for possible treatment of SARS-CoV-2.

### 3.4. Other Medications

#### 3.4.1. Umifenovir

Umifenovir (Arbidol) is an anti-influenza drug used in Russia and China with in vitro activity against SARS-CoV. It is thought to inhibit viral fusion, but its mechanism remains unclear [85]. While there is very limited data to support the use of Arbidol in SARS-CoV-2, initial retrospective observational data suggests that umifenovir decreases viral load more rapidly than does LPV/r (duration of positive RNA test 9.5 days umifenovir; 11.5 days LPV/r; *p* < 0.01), without significant adverse events [37,86].

#### 3.4.2. Indomethacin

Indomethacin is a non-steroidal anti-inflammatory drug (NSAID) that inhibits the cyclooxygenase (COX) enzymes; this inhibition decreases inflammation by lowering COX-mediated prostaglandin production. However, the use of NSAIDS has been controversial in the setting of COVID-19. NSAIDS improve outcomes in some viral respiratory infections, but they may mask symptoms and delay further treatment. Hypothetically, NSAIDS may increase viral uptake by increasing ACE2 receptor expression. Prior studies showed that indomethacin inhibits SARS-CoV RNA synthesis and blocks SARS-CoV replication in both Vero and human lung cell cultures [87]. Similarly, indomethacin inhibits SARS CoV-2 pseudovirus infection in monkey Vero cells [88]. While in vitro evidence suggests that indomethacin may offer therapeutic benefit in the setting of COVID-19, further studies are needed to clarify therapeutic safety and efficacy.

#### 3.4.3. Hydroxychloroquine/Chloroquine

The anti-malarial agent chloroquine and its analog, hydroxychloroquine, exert antiviral effects by multi-factorial mechanisms that include increasing intracellular vacuole pH, manipulating pathways involved in protein degradation, and modifying ACE2 receptor glycosylation [89,90]. In vitro studies of chloroquine for MERS-CoV showed inconsistent evidence of benefit [60]. Chloroquine has been researched extensively in vitro and in vivo for evidence of antiviral activity against SARS-CoV-2. Although in vitro studies of chloroquine and hydroxychloroquine were initially positive [91], early clinical results were inconsistent [92]. A recently published retrospective cohort study that compared treatment with hydroxychloroquine, azithromycin, hydroxychloroquine/azithromycin, or neither drug demonstrated no difference in inpatient mortality [93]. More statistically significant adverse effects, such as cardiac arrest, QT prolongation, diarrhea, and arrhythmias, were reported in patients treated with hydroxychloroquine. In light of the lack of benefit, the potential cardiac complications, and the risk of side effects, the USFDA revoked the Emergency Use Authorization for hydroxychloroquine and chloroquine for COVID-19 [94]. Furthermore, the Outcomes Related to COVID-19 treated with hydroxychloroquine among Inpatients with Symptomatic Disease (ORCHID) study was halted based on lack of benefit on interim analysis. Thus, at this time, there is no clear benefit to further evaluation of hydroxychloroquine [95].

#### 3.4.4. Chlorpromazine

Chlorpromazine hydrochloride, a dopamine receptor antagonist used as an antipsychotic and antiemetic, also inhibits the clathrin-mediated endocytosis pathway that SARS-CoV uses to enter cells [96]. In vitro studies showed that chlorpromazine strongly inhibits MERS-CoV and SARS-CoV [60], but it has not been used clinically in patients with MERS and SARS [96]. Its mechanism of action supports consideration as a potential candidate for treatment of SARS-CoV-2 infection [30,96]. The recent observation that the prevalence of severe COVID-19 is lower in patients receiving chlorpromazine has prompted further investigation into the use of this drug [97].

#### 3.4.5. Toremifene Citrate

Toremifene citrate is an estrogen receptor antagonist used to treat breast cancer. Its potential for antiviral repurposing has led to the discovery that toremifene destabilizes the Ebola virus membrane glycoprotein; this drug is thought to inhibit viral fusion and prevent viral replication [98,99]. Toremifene inhibits MERS-CoV and SARS-CoV infections in Vero cell cultures [43,100]. Since SARS-CoV-2 shares significant homology with these two viruses, this drug should be considered a potential therapeutic candidate for in vitro evaluation of antiviral activity against SARS-CoV-2. Recent analysis supports the use of toremifene as a rational candidate for testing in the context of SARS-CoV-2 [99].

#### 3.4.6. Loperamide

Loperamide is an opioid receptor agonist that is commonly used as an anti-diarrheal agent because it slows gut motility. It activates the mu opioid receptor, primarily within the gastrointestinal tract. Loperamide inhibits SARS-CoV and MERS-CoV replication in cell culture, though its anti-viral mechanism of action is not understood [101]. However, in light of its favorable safety profile and action in other coronaviruses, further investigation into the efficacy of loperamide as a therapy for SARS-CoV-2 infection is warranted.

## 4. Discussion

The novel pandemic COVID-19 is an emergent threat to global health [102]. COVID-19 is caused by a novel coronavirus that is structurally similar to the SARS-CoV virus that causes Severe Acute Respiratory Syndrome. There are currently no medications or vaccines proven to be effective for the treatment or prevention of SARS-CoV-2 [102]. SARS-CoV and MERS-CoV both belong to the *betacoronavirus* genus [3], and SARS-CoV-2 is highly homologous with SARS-CoV and moderately homologous with MERS-CoV [1,102]. This review builds on the premise that therapies with evidence for benefit in SARS-CoV and MERS-CoV infections are more likely to benefit patients with SARS-CoV-2 infections. The homology among these three viruses suggests opportunities for medication repositioning and rapid evaluation. Building on this previous research by using pre-existing candidate therapies will speed the process of testing and discovery. This review emphasizes those medications that have been approved for other indications and could be repurposed efficiently to treat SARS-CoV-2 (Table 1). Targeted enzyme inhibitors, broad-spectrum antivirals, and immunomodulators have important roles in treating viral infections, either as monotherapy or in combination [43]. The targeted antiviral enzyme inhibitors that have already shown particular promise are remdesivir, favipiravir, and the TMPRSS2 inhibitors such as camostat mesylate. These drugs are being investigated in clinical trials. Broad-spectrum antivirals, specifically ribavirin and interferons, warrant further investigation as part of adjunctive therapy. Although lopinavir/ritonavir combination therapy demonstrated no benefit in a recent clinical trial [28], it did decrease length of ICU stay. Thus, additional research may be warranted to determine the benefits and optimal use of this treatment option. For severe COVID-19 illness, the immunomodulators sarilumab, tocilizumab, and baricitinib may ameliorate the cytokine storm that contributes to mortality [48]. Convalescent plasma and humanized monoclonal antibodies should also be considered as short-term treatments that provide passive immunity.

Suggestions for further research include the repurposing and development of drugs that are thought to inhibit specific viral targets. The discovery of the structure of SARS-CoV-2 has provided essential information for drug targets and clinical trials [26,30,103]. Targeted enzyme inhibitors disrupt critical pathways. Disulfiram inhibits the MERS-CoV and SARS-CoV PLpro in vitro [35]. There are currently no in vivo studies of disulfiram, but further research may prove beneficial. Additionally, since Imatinib (Gleevec) inhibits viral replication by interfering with endosomal fusion in SARS-CoV and MERS-CoV [43,44], further study may be beneficial. Notably, inhibiting TMPRSS2 enzyme activity can prevent coronaviruses from entering host cells [30]. The TMPRSS2 inhibitors camostat mesylate and nafamostat mesylate, medications used in pancreatitis [30], and bromhexine, a mucolytic, are currently under review in clinical trials [34]. Some antibiotics, such as teicoplanin and dalbavancin, inhibit the activity of cathepsin L, which is involved in viral genome delivery [80,81]. These antibiotics may support combination therapy for SARS-CoV-2.

While an inspiring global research effort seeks to develop a vaccine against SARS-CoV-2, there are multiple existing therapies that are likely to benefit patients with COVID-19. This review summarizes currently available evidence for medications that are likely to be effective for treatment of SARS-CoV-2 based on past studies in the context of the highly homologous SARS-CoV and MERS-CoV. Extensive further research will be needed to identify the therapeutics that best treat patients with COVID-19.

## Figures and Tables

**Table 1:** Summary of pharmaceuticals with potential for repurposing for treatment of COVID-19 based on evidence in SARS or MERS. This table summarizes the class of each medication reviewed and briefly indicates the relevant mechanism of action. Numbers in the three columns “Evidence in SARS,” “Evidence in MERS,” and “Evidence in COVID-19” reference key citations that describe studies of each medication in the relevant disease (SARS, MERS, or COVID-19). The rightmost column, “Trials”, describes the number (n) of trials registered for each drug in the Cochrane Controlled Register of Trials as of 24 June 2020.

Class	Medication	Mechanism of Action	Evidence in SARS		Evidence in MERS		Evidence in COVID-19		Trials (*n*)
			In Vitro	In Vivo	In Vitro	In Vivo	In Vitro	In Vivo	
**Enzyme Inhibitors**	Remdesivir	RNA dependent RNA polymerase inhibitor	[4,10]	[4,10]	[4,10]	[4,11]	[4,9,12]	[13,14]	13
	Favipiravir	RNA dependent RNA polymerase inhibitor					[12,15]	[17]	19
	Oseltamivir	Interferes with release of viral progeny from infected host cells						[20]	3
	Lopinavir/Ritonavir	Inhibits viral protease enzyme. Ritonavir also inhibits metabolism of lopinavir		[24,25]		[24]	[27]	[28,29]	27
	Camostat	Inhibits TMPRSS2 enzyme	[30,31]		[30,31]				2
	Nafamostat	Inhibits TMPRSS2 enzyme			[32]		[33]		3
	Bromhexine	Inhibits TMPRSS2 enzyme							2
	Disulfiram	Inhibits hepatic aldehyde dehydrogenase; inhibits PLpro	[35]		[35]				0
	*N*-(2-Aminoethyl)-1-aziridineethanamine	ACE2 Inhibitor (preclinical)	[40]						0
	Imatinib	Inhibits tyrosine kinase	[43,44]		[43,44]				3
	Baricitinib	Inhibits JAK1 and AAK1 kinases, interferes with viral endocytosis, blunts cytokine storm						[47]	4
	Ribavirin	Inhibits inosine monophosphate dehydrogenase enzyme	[49]	[51]		[50]		[52]	6
**Immuno-modulators**	Convalescent Plasma	Antibodies target SARS-CoV-2		[55]				[56,57,58]	111
	mAb 4C2h	Neutralizes spike receptor binding domain of MERS-CoV (preclinical)				[60]			0
	mAb 5H10	Neutralizes spike receptor binding domain of SARS-CoV (preclinical)		[61]					0
	Tocilizumab	Humanized IL-6 receptor antibody						[62]	21
	Sarilumab	Humanized IL-6 receptor antibody						[63]	13
	Interferons	Activates host immune system	[65,66]	[64,66]	[66]	[66]	[66]	[52,68]	49
	Cyclosporine A	Calcineurin inhibitor	[70]			[70]		[73]	3
	Mycophenolic Acid	Immunosuppressant; may inhibit PLpro	[45]		[45]				0
	Methylprednisolone	Suppresses host inflammatory responses		[75,76]		[75,76,77]		[76,77]	10
	Dexamethasone	Suppresses host inflammatory responses		[75,76]		[75,76]		[76,78]	11
**Antibiotics**	Teicoplanin	Inhibits cathepsin L-mediated spike cleavage			[80]		[80]	[80,82]	1
	Dalbavancin	Unclear	[81]		[81]				0
	Oritavancin	Unclear	[81]		[81]				0
	Nitazoxanide	Uncouples oxidative phosphorylation			[37,84]				14
**Other Medications**	Umifenovir	Unclear	[85]					[37,86]	4
	Indomethacin	NSAID; inhibits COX	[87]				[88]		1
	Hydroxychloroquine/Chloroquine	Increases endosomal pH of phagolysosome interferes with viral fusion with cell; modifies ACE2 receptor; modifies protein degradation pathways			[60]		[91]	[92,93,95]	>150
	Chlorpromazine	Dopamine receptor antagonist; inhibits clathrin-mediated viral endocytosis	[60]		[60]			[97]	2
	Toremifene Citrate	Estrogen receptor antagonist	[43,100]		[43,100]				0
	Loperamide	Mu opioid receptor agonist	[101]		[101]				0

## Abbreviations

AAK-1	Adaptor-associated kinase
Abl2	Abelson tyrosine-protein kinase 2
ACE	Angiotensin-converting enzyme
ACE2	Angiotensin-converting enzyme 2
ACTT-1	Adaptive COVID-19 Treatment Trial
COVID-19	Coronavirus disease 2019
COX	Cyclooxygenase
HIV	Human immunodeficiency virus
ICU	Intensive care unit
IL-6	Interleukin 6
JAK	Janus kinase
LPV/r	Lopinavir/ritonavir
MERS-CoV	Middle East respiratory syndrome coronavirus
MPA	Mycophenolic acid
NAAE	*N*-(2-aminoethyl)-1-aziridineethanamine
NSAID	Non-steroidal anti-inflammatory drug
NTZ	Nitazoxanide
ORCHID	Outcomes Related to COVID-19 treated with hydroxychloroquine among Inpatients with symptomatic Disease
PLpro	Papain-like protease
RdRp	RNA-dependent RNA polymerase
RDV-TP	Remdesivir triphosphate
RECOVERY	Randomised Evaluation of COVID-19 therapy
RSV	Respiratory Syncytial Virus
S protein	Spike protein
SARS-CoV	Severe acute respiratory syndrome coronavirus
SARS-CoV-2	Severe acute respiratory syndrome coronavirus 2
TMPRSS2	Transmembrane protease, serine 2
USFDA	U.S. Food and Drug Administration
